# Climate change adaptation strategies and production efficiency: The case of citrus farmers in the Limpopo province, South Africa

**DOI:** 10.4102/jamba.v13i1.1093

**Published:** 2021-11-29

**Authors:** Samuel Joseph, Michael A. Antwi, Clarietta Chagwiza, Theresa T. Rubhara

**Affiliations:** 1 Department of Agriculture and Animal Health, Faculty of Agriculture and Environmental Sciences, University of South Africa, Johannesburg, South Africa

**Keywords:** climate change, adaptation strategies, stochastic frontier production function, profit efficiency, technical efficiency

## Abstract

Climate change adaptation policies and strategies have inevitably become an integral component of agricultural production on a global scale. The evaluative extent to which these adaptation techniques have influenced agricultural productivity is inherently exiguous. Citrus production in tropical regions such as South Africa, is more vulnerable to climate change as the region already experience hot and dry climate, hence the need to implement different strategies for climate change adaption in these regions. This study was designed to assess the effect of adopting the following climate change adaptation measures: planting drought resistant varieties, rainwater harvesting, planting early maturing varieties, integrated pest management (IPM) , changing fertiliser type, and applying drip irrigation to manage climate challenges on the production efficiency of citrus farmers in the Limpopo province of South Africa. The stochastic frontier production function with Cobb Douglas production functional form was used to analyse the productivity of farmers’ vis-à-vis adopted climate change strategies. A survey was conducted and data were collected through a semi-structured questionnaire administered to respondents from 235 production units in the five district municipalities of Limpopo. The likelihood ratio tests for profit models showed that farmers were profit efficient considering the identified adaptation strategies. The variables that influenced profit efficiency was price of fertiliser (*p* < 0.010) and water cost (*p* < 0.010). The inefficiency model showed that besides changing fertiliser as an adaptation measure, the other adaptation strategies including IPM, water harvesting and planting drought resistant varieties did not change the profit efficiency of farmers. Therefore, the results indicate that citrus farmers can still adapt to climate change and remain profit efficient.

## Introduction

Climate change substantially defines agricultural productivity as it influences several input balances, which support the entire agricultural system. The impacts of climate change are manifested in many ways, which include prolonged periods of moisture stress, high incidence of pests and diseases, increased salinity, high temperatures and floods (Khanal et al. 2018a). Global moisture stress and temperature variation have severe effects on world food production (Khanal et al. 2018b). The Integrated Panel on Climate Change (IPCC 2013) predicted an increase in temperature of between 0.8 and 4 degrees in the 21st century. In perennial crops such as citrus fruits, high temperatures may affect the budding of flowers in winter whereas during the fruit development phase, incidences of high temperature and moisture stress compromises fruit maturation and expansion (De Ollas et al. 2019), thus impacting negatively the overall quantity and quality of fruits. The increase in atmospheric carbon dioxide concentration has also been found to increase the incidence of plant pests and diseases, thus indirectly reducing yield (Rajatiya et al. 2018). Various governments and international development organisations have begun to develop strategies to adapt to the ravaging effects of climate change (UNDP 2007). The United Nations Development Plan (UNDP) initiative of the climate adaptation programme has a well-defined framework, which is structured to oversee implementation of the adaptation programme of countries and regions to climate change (UNDP 2007). There is notable distinctiveness in the experiences of farmers at the district level, which often result in different adaptation needs and uncoordinated action within households (Paavola & Adger 2005). The major advantage of pursuing farm level adaptation planning is that small organisations can move quickly to create adaptation strategies, which will directly benefit their communities (Picketts, Curry & Rapaport 2012).

### Citrus production in South Africa

Approximately 100 000 people are employed in the citrus value chain in South Africa, therefore the industry is considered significant in employment creation (DAFF 2017). In the 2016–2017 season, the industry represented 25% of the total horticultural production and contributed R19.1 billion to the total gross value (DAFF 2018). The industry is also a major foreign currency earner. According to DAFF (2017, the Limpopo province stands as the leading grower of citrus in South Africa, producing 42% of the total national citrus output, cultivated on over 26 960 hectares of land. The province is characterised by threatening climate challenges with previous studies indicating that farmers in the area acknowledge that the province is becoming warmer and drier (Maponya 2012). Farmers in the province are also aware of higher incidences of pests, increases in annual temperature and drought, necessitating increased engagements in various adaptation techniques in previous decades (Maponya 2012). The government has launched various strategic efforts in mitigating the impact on agriculture and livelihoods as indicated by the Limpopo Department of Economic Development, Environment and Tourism (LEDET) Climate Change Response Strategy (2008, 2016). The Limpopo government’s response to the climate affront has propelled a five-point agenda on climate change management initiative with focus on the following areas: agriculture, water supply, ecosystems (terrestrial and aquatic), human health livelihoods and settlements. The researchers’ motivation for this study, thus aligns mainly with agriculture and water supply. The Limpopo provincial government aims to ensure that proposed water-related infrastructure projects explicitly integrate climate change resilience into their planning and design stages (LEDET 2008). Farm level adaptation strategies listed in the provincial plan include: plant management, integrated pest management (IPM) and soil and water conservation techniques. The citrus industry is unique because it mainly depends on irrigation, therefore drip irrigation and rainwater harvesting were some of the adaptation strategies specified for the citrus industry (LEDET 2016). The concept of agricultural adaptation addresses both arms of ‘vulnerability’ and ‘resilience’ as they affect food systems, which are primordially economic, social and ecological in context (Ericksen 2008; Füssel & Klein 2006; Meze-Hausken 2004).

### Agriculture production efficiency and climate change adaptation in Africa

The following common adaptation strategies were identified from the literature with regard to crop production in Africa: the use of drought resistant varieties, crop diversification, soil moisture conservation, changes in planting dates and improved irrigation (Akinnagbe & Irohibe 2014; Kuwornu et al. 2013). Similarly, Debela et al. (2019) explored the adaptation strategies employed by pastoral and agro pastoral farmers in Southern Ethiopia, and the findings revealed that farm practice adjustments and diversification to non-pastoral ventures were the most prominent adaptation strategies identified amongst farmers in the region. On the other hand, Ajibefun, Battese and Daramola (2002); Marinda, Bangura and Heidhues, (2006) used Cobb Douglas function to ascertain factors affecting technical efficiency in agricultural production. The factors age, experience, education, household labour usage ratio, access to credit, fertiliser use and distance from the farm to the main road were found to be significant (Ajibefun et al. 2002; Marinda et al. 2006). A review by Zwane (2019) explained that hydrological drought emanating from a reduction in rainfall and surface water resources has the major detrimental effects in citrus production in South Africa. Hydrological drought implies there is not enough water for irrigation therefore there won’t be enough water for each production stage for the crops. For instance, Swart (2016) reported that the 2015–2016 drought experienced in South Africa had long-term effects for the citrus industry as water reserves were diminished.

Although previous studies identified various existing climate change adaptation strategies amongst farmers in different regions few of these studies have considered the quantitative assessment of the efficiency and influence of adaptation strategies on agricultural productivity. Khanal et al. (2018a) used the endogenous switching regression model and found that soil and water management practices had the most significant positive impact on food productivity in Nepal. Roco et al. (2017) used the Stochastic Frontier Approach in Chile to realise that climate adaptive practices such as irrigation, improved annual crops’ production efficiency. Khanal et al. (2018a) and Roco et al. (2017) provided insights on climate change adaptation and production efficiency, however the scope was limited to annual crops only.

Empirical evidence on impacts of climate change for the citrus crops in South Africa is relatively scarce. In Limpopo, farmers are already engaging in various measures towards adapting to the climate inconstancy, however, it is unclear how resilient these interventions would be in the face of extreme climate change complexities (Vermeulen et al. 2010). Empirical evidence also shows that although farmers have embraced adaptation strategies to counter the impacts of climate change, such interventions come at a cost (Khanal et al. 2018a; Rajatiya et al. 2018), which is likely to impact the profit and technical efficiency of crop production (Roco et al. 2017). The objective of this study is, therefore, to analyse the influence of drip irrigation, rainwater harvesting, IPM, use of drought resistant varieties, and changes in type of fertiliser used on production efficiency of citrus farmers in the Limpopo province, South Africa. The results from the study will help in providing relevant insights into profitable agricultural production for the citrus industry.

## Materials and methods

### Description of the study area

**FIGURE 1:**
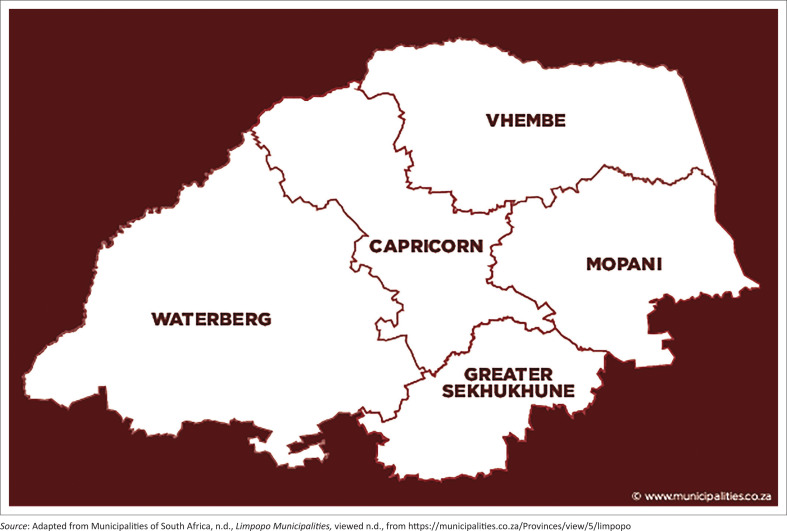
Map of the Limpopo province.

The study was carried out in the Limpopo province of South Africa (see Figure 1), from September 2017 to February 2018. The Limpopo province lies on a land expanse of about 125 754 km^2^ representing close to 10.4 % of the entire topography of South Africa. It has a population of approximately 5.4 million people with 55% being women (Stats SA 2016). The citrus farmers in the area consist mainly of commercial farmers, although smallholder farmers are also available. The province is characterised by numerous environmental and topographical features. The northern terrain comprises the Limpopo plain and the Bushveld basin surrounded by the Central Highland, bordered to the east by both the Great Escarpment and the Eastern Plateau slope (Stats SA 2016). The province accounts for the largest production of citrus in South Africa (DAFF 2017). Besides other climatic variables that may include temperature range and humidity, rainfall is a critical factor in determining the success of agricultural production. Malherbe, Engelbrecht and Landman (2012) observed that noticeable decline has been witnessed in the total rainfall recorded in most part of the Limpopo River Basin and eastern part of Southern Africa generally. South African Weather Service (SAWS) (2017) report indicated that an observed period between 1904 and 2016 showed early heavy rainfall between 1904 and 1920, and this trend declined over the years on an average of about –6%. Projections on climate by SAWS (2017) also strongly suggest that annual total rainfall will continually decrease towards a low percentage of about –15% towards 2066–2095.

### Research design

The Limpopo province was purposively sampled as the study area because the province is the number one citrus producer in the country, therefore, it has a large number of farmers. Citrus farms across the study area are called Production Unit Code (generically coded as PUCs). According to the statistics provided by the Limpopo Department of Agriculture, there were approximately 598 production units in 2017. The population consisted of PUCs across the five district municipalities in the Limpopo province comprising both commercial and smallholder farmers. The Krejcie and Morgan (1970) formula was used as shown here in Equation 1 to come up with a sample size of 235.


$$S=\frac{x^{2}NP(1-P)}{d^{2}(N-1)+x^{2}P(1-P)}$$ [Eqn 1]

where:

*S* = required sample size

= chi-square of degree of freedom 1 and confidence interval 95% (which is 3.841 for 95% confidence level)

*N* = population size of 598

*p* = population proportion assumed to be 0.5

*d* = margin of error assumed to be 0.05

The random sampling technique was used to select the respondents and data were collected by trained enumerators using semi-structured questionnaires in the five district municipalities, namely Capricorn, Mopani, Sekhukhune, Vhembe and Waterberg. Fifteen of the questionnaires were not included in the data analysis as they had some data gaps, which were realised at the data cleaning phase; hence the remaining 220 PCUs were considered for the research. Data collected included quantities of inputs, household demographics and climate change adaptation strategies employed by citrus farmers.

Descriptive and inferential statistics such as mean, percentages, frequencies, standard deviation and probabilities were used in analysing the data. The Statistical Package for the Social Sciences (SPSS) version 24 was used for the descriptive statistics, whilst Stata version 14 was used for inferential data analysis.

### Model specification

Production efficiency is analysed by two components: technical and allocative efficiency. Two main econometric measures used to model efficiency are: the Data Envelope Analysis and the Stochastic Frontier Model (Mafuse et al. 2021; Parman & Featherstone 2019). The analytical approach in this study adopts the Stochastic Frontier Production Function model and the theoretical assumption of the functional form was the Cobb Douglas production function. The choice of the model was influenced by literature as this stochastic model is considered a reliable method in estimating efficiency in production (Roco et al. 2017). The profit function approach combines the concepts of technical and allocative efficiency in the profit relationship and farm is assumed to be optimising profits (Kaka et al. 2016). Therefore, a farm is considered to be efficient if it is able to use inputs in optimal proportions given their respective prices (Shanmugam & Venkataramani 2006). As suggested by Aigner, Lovell and Schmidt (1977), the Stochastic frontier profit model is presented as Equation 2:


$$\text{ln}\prod_{i}=\alpha_{0}++\sum \alpha_{i}\text{ln}(X_{i})+(c)$$ [Eqn 2]

where:

*α_o_* = parameters estimate

*V_i_* = two-sided standardised random error

*U_I_* = inefficiency variables

*Π_i_* = gross revenue from citrus production in Rands

*X*_1_ = cost of labour in Rands

*X*_2_= cost of rent in Rands

*X*_3_= fertiliser cost in Rands

*X*_4_= cost of agrochemicals in Rands

*X*_5_ = current price of fixed assets in Rands

Whilst the profit inefficiency effect is expressed as Equation 3:


$${\text{U}}_{i}=\delta_{0}++\sum_{i=1}^{8}\delta_{i}\lambda_{ij}$$ [Eqn 3]

where:

*U_i_* = inefficiency variables (*climate change adaptation strategies*)

*δ_i_* = coefficients of adaptation strategies

*λ_ji_* = adaptation strategies

*λ*_1_ = cultivated drought resistant varieties

*λ*_2_ = cultivated early maturing varieties

*λ*_3_ = integrated pest management

*λ*_4_ = constructed dams/harvested rainwater

*λ*_5_ = dam construction/water harvesting

*λ*_6_ = diversification of varieties

*λ*_7_ = applied drip irrigation

The inefficiency variables represent the climate change adaptation variables in citrus production as extracted from literature (Kuwornu et al. 2013; Roco et al. 2017). The variables were recorded as dummy represented as 0 where farmers did not adopt the specific climate change adoption strategy and 1 where farmers adopted it.

### Ethical considerations

Ethical clearance was obtained from the University of South Africa, National Health Research Ethics Councils, reference number: REC-170616-051.

## Results and discussion

### Demographic and socio-economic characteristics of respondents

Table 1 presents the demographic and socio-economic characteristics of citrus famers in the study. Female farmers constituted about 4.1% of the total population whilst 95.9% were male. About 63.9% of the farmers were aged between 36 and 55 years, 32.2% were between 56 and 75 years, whilst 32.2% were aged between 56 and 75 years. This shows that the citrus industry labour is spread across all the age groups. The analysis further shows that about 76.8% of the farmers had from 10 to 200 hectares of citrus farmland, 20.6% had between 201 and 400 hectares, 1.8% had farms within the range of 401–600 hectares, whilst 0.5% had between 601 and 800 hectares.

**TABLE 1:** Demographic and socioeconomic characteristic of respondents.

Variable	Frequency (*n* = 220)	Percentage
**Gender of farmers**		
Female	9	4.1
Male	211	95.5
**Age of farmers**		
≤ 35	8	3.9
36–55	141	63.9
56–75	71	32.2
**Farm size in hectares**		
≤ 200	169	76.8
201–400	46	20.9
401–600	4	1.8
601–800	1	0.5
**Farming experience of respondents in years**		
≤ 10	9	4.1
11–20	72	33.0
21–30	108	49.0
31–40	29	13.0
41–50	2	0.9
**Types of citrus cultivated by farmers**		
Grapefruit, valencia, navel	21	9.5
Lemon, navel, valencia, grapefruit	28	12.7
Grapefruit, valencia, lemon	152	69.1
Grapefruit, valencia	12	5.5
Navel, valencia, lemon	7	3.2
**Source of climate change adaptation strategies**		
Citrus Research International (CRI)	195	88.6
Experience	15	6.8
Extension officers	8	3.6
Media	2	0.9

Majority of the respondents (69.1%) cultivated grapefruit, valencia and lemon; 7% cultivated lemon, navel, valencia and grapefruit, 9.5% cultivated grapefruit, valencia and navel. About 5.5% planted grapefruit and valencia, whereas 3.2% planted navel, valencia and lemon. The findings presented in Table 2 also reveal that 4.1% of the respondents had 10 years or less of citrus farming experience, 33% of them had between 11 and 20 years of experience. Majority of the respondents (49%) had between 21 and 30 years’ experience, 13% have been citrus farmers for at least 31 years and at most 40 years whilst 0.9% of them had been in the industry for between 41 and 50 years. The results indicate that 88.6% of the respondents sourced global climate change resources on adaptive measures from Citrus Research International (CRI), 6.8% relied on experience, close to 3.6% from trusted extension officers whilst the rest (0.9%) relied on the media.

**TABLE 2:** Climate change adaptation strategies adopted by citrus farmers.

Adaptation strategies	Frequency (*n*)	Percentage
Planted drought resistant variety	62	28.2
Planted early maturity variety	32	14.5
Changed type of fertiliser	16	7.3
Applied IPM	203	92.3
Dams/harvested water/access to water right	192	87.3
Variety diversification	34	15.5
Applied drip irrigation	173	78.6

IPM, integrated pest management.

### Climate change adaptation strategies employed by citrus farmers

Citrus farmers in the province have adopted a variety of climate change adaptation strategies. The strategies are not implemented in isolation as a farmer can use two or more strategies simultaneously. Integrated pest management (92.3%), water harvesting (87.3%) and drip irrigation (78.6%) were adapted by a larger percentage of the population. As highlighted by Zwane (2019), hydrological drought is one of the effects of climate change, which has detrimental effects on citrus production, hence farmers are likely to implement water harvesting and drip irrigation strategies. The high frequency of people having either access to water rights, dams or using water harvesting techniques can also be attributed to the institutional support as highlighted in the LEDET (2016) strategy. Maponya (2012) also indicated that citrus farmers were aware of high incidences in pest and diseases hence the high levels of IPM amongst the farmers. In contrast to previous studies that showed high adaptation rates for drought resistant varieties in maize farmers in Africa (Acevedo et al. 2020; Lunduka et al. 2019), this climate change adaptation strategy was used by only 28% of the citrus farmers in the Limpopo province. The least implemented practice (7%) was the use of different chemical fertilisers by farmers. This could be attributed to the fact that changing fertilisers can be costly for the farmers, hence if farmers consider the adaptation to be expensive, they are less likely to practice that strategy.

### Estimates for the stochastic frontier parameters for the profit function

We compared two models for fitness to analyse the data: model 1 (restricted) and model 2 (unrestricted). The fitness comparison was achieved through the likelihood ratio test of hypothesis for model fitness. As indicated in the results presented in Table 3, model 1 was nested in model 2, with chi-squared value of 7.77 and the degree of freedom 7, equivalent to the number of restricted parameters. The *p*-value derived for the chi-square was greater than 0.05, therefore we failed to reject the null hypothesis H_0_; that citrus farmers in the study area are fully profit efficient. In analysing the data, model 1 (restricted), in which the inefficiency variables were absent is most preferable. Therefore, the discussion of results in this analysis is based on the restricted model 1.

**TABLE 3:** Likelihood ratio test of hypothesis for profit efficiency model.

Citrus farmers	Model 1	Model 2
Log likelihood	−165.096	−162.978
***N***	220	220
Assumption	Model 1 nested in model 2	-
Likelihood ratio chi^2^(7)	7.77	-
Probability > chi^2^	**0.3537**	-

Decision: Probability > chi^2^ = 0.3537 > 0.05; fail to reject null hypothesis that citrus farmers are fully profit efficient.

### The profit function model

The maximum likelihood estimates measures for the two evaluated profit models are shown in Table 4. The variables: price of fertiliser (*z* < 0.01) and cost of water (*p* < 0.01) were found to be statistically significant in the adopted model with positive coefficients. This implies that increase in the costs of fertiliser and water resources would potentially inflate output value by 38% and 37%, respectively. The inefficiency model that explains the influence of adaptation strategies showed that change in fertiliser type would result in profit inefficiency (*p* < 0.1). In order to adapt to climate change impacts, farmers are advised to use high nitrogen fertilisers for increased yields, however such shifts can be costly. According to Joseph et al. (2020), one of the challenges to adaptation practises in citrus farmers in South Africa was the high cost of adaptation. Khanal et al. (2018a) found water management activities as significantly improving food production in Nepal, however, in this study, although the coefficients of water harvesting and drip irrigation are both positive, they are not statistically significant. Planting drought resistant varieties, early maturing varieties, variety diversification and IPM were also not statistically significant. This implies that farmers can still practise the other six climate adaptation practises included in the model besides change of fertiliser and remain technically efficient. The results are consistent with findings from the study by Khanal et al. (2018b) that the cost of adaptation will always be lower than the loss because of not adopting any climate change adaption strategies completely.

**TABLE 4:** The Maximum Likelihood Estimates (MLE) results of the stochastic frontier profit function and adaptation strategies for citrus farmers in the Limpopo province.

Model	Model (1)				Model (2)		
	Variable	Coefficient	Standard error	*p* > |*z*|	Coefficient	Standard error	*p* > |*z*|
**Model (Profit)**							
Constant	*α*_0_	1.476	0.720	0.041**	1.291	0.729	0.077*
Ln (Agrochemical) (*X*_1_)	*α*_1_	0.114	0.139	0.410	0.152	0.139	0.276
Ln (Cost of labour) (*X*_2_)	*α*_2_	0.146	0.124	0.241	0.124	0.122	0.311
Ln (Farmland rent) (*X*_3_)	*α*_3_	-0.002	0.006	0.704	-0.003	0.006	0.621
Ln (Price of fertilisers) (*X*_4_)	*α*_4_	0.380	0.103	0.000***	0.386	0.102	0.000***
Ln (Cost of electricity) (*X*_5_)	*α*_5_	0.159	0.117	0.171	0.167	0.116	0.152
Ln (Cost of water) (*X*_6_)	*α*_6_	0.365	0.060	0.000***	0.343	0.061	0.000***
**Profit Inefficiency Model**							
Constant	*λ*_0_	0	-	-	-0.915	0.526	0.082*
Drought resistant variety	*λ*_1_	0	-	-	0.135	0.498	0.786
Improved/early maturing cultivars	*λ*_2_	0	-	-	0.400	0.552	0.469
IPM	*λ*_3_	0	-	-	-0.285	0.472	0.545
Change fertiliser type	*λ*_4_	0	-	-	-1.003	0.608	0.099*
Dam/harvesting rainwater	*λ*_5_	0	-	-	-0.146	0.431	0.735
Variety diversification	*λ*_6_	0	-	-	-0.395	0.660	0.549
Applied drip irrigation	*λ*_7_	0	-	-	0.357	0.400	0.372
Prob > chi^2^		0.000	-		0.000	-	
Log likelihood		-165.978	-		-162.096	-	
***N***		220	-		220	-	

Assumption: Model 1 nested in Model 2. Likelyhood ratio chi^2^ (7) = 7.77, Probability > chi^2^ = **0.3537**; Failed to reject null hypothesis.

IPM, integrated pest management.

* , *p* < 0.10;

** , *p* < 0.05;

*** , *p* < 0.01.

## Conclusions

This study investigated the influence of climate change adaptation strategies on citrus production in the Limpopo province, South Africa. Some of the climate change adaptation techniques that farmers adopted include water harvesting techniques, planting drought resistant varieties and IPM. The likelihood ratio tests showed that the model without the inefficiency effects of adaptation strategies for the profit efficiency best explained the data. This implies that citrus farmers in the study area were profit efficient considering the climate change adaptation measures associated with production activities in the study area. As this study reveals that the citrus farm is profit efficient and variables such as fertiliser and water cost significantly increased output value, it implies that farmers are using the water and fertiliser profitably. The adaption strategy of changing fertiliser to another with more nitrogen content can be costly to farmers resulting in inefficiencies. In other words, farmers who opt to change their fertilisers ceteris paribus are likely to have reduced profits. In this context, farmers should be encouraged to adopt climate change practices such as water harvesting, planting drought resistant varieties and IPM because such practices do not negatively impact farm profits. Initiatives should be directed at improving water access to citrus farmers. Construction of more dams and effective water management policies would go a long way in ensuring water availability and sustainability for agricultural purposes in the province.

## References

[CIT0001] Acevedo, M., Pixley, K., Zinyengere, N., Meng, S., Tufan, H., Cichy, K. et al., 2020, ‘A scoping review of adoption of climate-resilient crops by small-scale producers in low- and middle-income countries’, *Nature Plants* 6, 1231–1241. 10.1038/s41477-020-00783-z 33051616 PMC7553851

[CIT0002] Aigner, D.J., Lovell, C.A.K. & Schmidt, P., 1977, ‘Formulation and estimation of stochastic production function models’, *Journal of Econometrics* 6(1), 21–37.

[CIT0003] Ajibefun, I.A., Battese, G.E. & Daramola, A.G., 2002, ‘Determinants of technical efficiency in smallholder food crop farming: Application of stochastic frontier production function’, *Quarterly, Journal of International Agriculture* 41(3), 225–240.

[CIT0004] Akinnagbe, O. & Irohibe, I., 2014, ‘Agricultural adaptation strategies to climate change impacts in Africa: A review’, *Bangladesh Journal of Agricultural Research* 39(3), 407–418.

[CIT0005] Battese, G.E. & Coelli, T.J., 1995, ‘A Model for technical inefficiency effect in stochastic frontier production functions for panel data’, *Empirical Economics* 20(1995), 325–332.

[CIT0006] Debela, N., Mcneil, D., Bridle, K. & Mohammed, C., 2019, ‘Adaptation to climate change in the pastoral and agropastoral systems of Borana, South Ethiopia: Options and barriers’, *American Journal of Climate Change* 08(01), 40–60.

[CIT0007] De Ollas, C., Morillón, R., Fotopoulos, V., Puértolas, J., Ollitrault, P., Gómez-Cadenas, A. & Arbona, V., 2019, ‘Facing climate change: Biotechnology of iconic Mediterranean woody crops’, *Frontiers in Plant Science* 10, 427. 10.3389/fpls.2019.00427 31057569 PMC6477659

[CIT0008] Department of Agriculture, Forestry and Fisheries (DAFF), 2017, *A profile of the South African citrus market value chain*, viewed from https://www.nda.agric.za/doaDev/sideMenu/Marketing/Annual%20Publications/Commodity%20Profiles/field%20crops/Citrus%20Market%20Value%20Chain%20Profile%2020.

[CIT0009] Department of Agriculture, Forestry and Fisheries (DAFF), 2018, *A profile of the South African citrus market value chain*, viewed 13 January 2020, from http://www.nda.agric.za/doaDev/sideMenu/Marketing/Annual%20Publications/Commodity%20Profiles/Citrus%20Market%20Value%20Chain%20Profile%202018.pdf.

[CIT0010] Ericksen, P.J., 2008, ‘Conceptualizing food systems for global environmental change research’, *Global Environmental Change* 18(1), 234–245.

[CIT0011] Füssel, H.-M. & Klein, R.T.J., 2006, ‘Climate change vulnerability assessments: An evolution of conceptual thinking’, *Climate Change* 75(2006), 301–329.

[CIT0012] IPCC, 2013, *Causes of climate change impacts and vulnerability, responses strategies, mitigation and adaptation*, Working Group, 1 Contribution to the IPCC Fifth Assessment Report, Climate Change: The Physical Science Basis.

[CIT0013] Joseph, S., Rubhara, T.T., Antwi, M.A. & Oduniyi, O.S., 2020, ‘Challenges to climate change adaptation practices among citrus farmers in Limpopo Province, South Africa’, *The International Journal of Climate Change: Impacts and Responses* 12(3), 19–32. 10.18848/1835-7156/CGP/v12i03/19-32

[CIT0014] Kaka, Y., Shamsudin M.N., Radam, A., Ismail A.D. & Latif, I.A., 2016, ‘A profit efficiency among paddy farmers: A cobb-douglas stochastic frontier production function analysis Yahaya’, *Journal of Asian Scientific Research* 6(4), 66–75.

[CIT0015] Khanal, U., Wilson, C., Hoang, V.N. & Lee, B.L., 2018a, ‘Farmers’ adaptation to climate change, its determinants and impacts on rice yield in Nepal’, *Ecological Economics* 144(2018), 139–147.

[CIT0016] Khanal, U., Wilson, C., Lee, B.L. & Hoang, V., 2018b, ‘Climate change adaptation strategies and food productivity in Nepal: A counterfactual analysis’, *Climate Change* 148(4), 575–590.

[CIT0017] Krejcie, R.V. & Morgan, D.W., 1970, ‘Determining sample size for research activities’, *Educational and Psychological Measurement* 1970(30), 607–610.

[CIT0018] Kuwornu, J.K.M., Al-hassan, R.M., Etwire, P.M. & Osei-Owusu, Y., 2013, ‘Adaptation strategies of smallholder farmers to climate change and variability: Evidence from Northern Ghana’, *Information Management and Business Review* 5(5), 233–239.

[CIT0019] Limpopo Department of Economic Development, Environment and Tourism (LEDET), 2008, Proceedings of the International Climate Change Conference, Oasis Lodge, Polokwane, South Africa, 20–25 October 2008, Department of Forestry, Fisheries and the Environment, Pretoria.

[CIT0020] Limpopo Department of Economic Development, Environment and Tourism (LEDET), 2016, *Provincial climate change response strategy 2016–2020*, viewed 15 July 2021, from http://policyresearch.limpopo.gov.za/bitstream/handle/123456789/1251/Limpopo_ClimateChange_Response_Strategy__2016_2020_Final_%284.

[CIT0021] Limpopo Provincial & District Municipality Maps, (n.d), viewed 16 August 2021, from www.municipalities.co.za.

[CIT0022] Lunduka, R.W., Mateva, K.I., Magorokosho, C. & Manjeru, P., 2019, ‘Impact of adoption of drought-tolerant maize varieties on total maize production in south Eastern Zimbabwe’, *Climate and Development* 11(1), 35–46. 10.1080/17565529.2017.1372269 30881484 PMC6397629

[CIT0023] Mafuse, N., Mushunje, A., Tatsvarei, S. & Zivenge, E., 2021, ‘Two stage maize supply chain model for production and marketing efficiency’, *International Journal of Agricultural Science, Research and Technology in Extension and Education Systems* 11(1), 21–32.

[CIT0024] Malherbe, J., Engelbrecht, F. & Landman, W.A., 2012, ‘Projected changes in tropical cyclone climatology and landfall in the Southwest Indian Ocean region under enhanced anthropogenic forcing’, *Climate Dynamics* 40(2013), 2867–2886.

[CIT0025] Maponya, K., 2012, ‘Climate change and agricultural production in Limpopo province: Impacts and adaption options’, A thesis submitted in accordance with the requirements for the degree of Doctor of Philosophy in the subject Environmental Science at the University of South Africa.

[CIT0026] Marinda, P., Bangura, A. & Heidhues, F., 2006, ‘Technical efficiency analysis of male and female-managed farms: a study of maize production in West Pokot District, Kenya’, Poster paper prepared for presentation at the International Association of Agricultural Economists Conference, Gold Coast, Australia, August 12–18, 2006.

[CIT0027] Meze-Hausken, E., 2004, ‘Contrasting climate variability and meteorological drought with perceived drought and climate change in northern Ethiopia’, *Climate Research* 27(1), 9–31.

[CIT0028] Municipalities of South Africa, n.d., *Limpopo Municipalities*, viewed n.d., from https://municipalities.co.za/Provinces/view/5/limpopo

[CIT0029] Paavola, J. & Adger, T.W., 2005, ‘Analysis of fair adaptation to climate change’, *Ecological Economics* 56(2006), 594–609.

[CIT0030] Parman, B.J. & Featherstone, A.M., 2019, ‘A comparison of parametric and nonparametric estimation methods for cost frontiers and economic measures’, *Journal of Applied Economics* 22(1), 60–85. 10.1080/15140326.2018.1526868

[CIT0031] Picketts, I.M., Curry, J. & Rapaport, E., 2012, ‘Community adaptation to climate change: Environmental planners’ knowledge and experiences in British Columbia, Canada’, *Journal of Environmental Policy and Planning* 14(7), 119–137.

[CIT0032] Rajatiya, J., Varu, D.K., Gohil, P., Solanki, M., Halepotara, F., Gohil, M. et al., 2018, ‘Climate change: Impact, mitigation and adaptation in fruit crops’, *International Journal of Pure and Applied Biosciences* 6(1), 1161–1169.

[CIT0033] Roco, L., Bravo-Uret, B., Engler, A. & Jara-Rojas, R., 2017, ‘The impact of climatic change adaptation on agricultural productivity in Central Chile: A stochastic production frontier approach’, *Sustainability* 9(9), 1–16.

[CIT0034] South African Weather Service (SAWS), 2019, *Annual climate summary for South Africa 2018*, Department of Forestry, Fisheries and the Environment, Pretoria.

[CIT0035] Shanmugam, K.R. & Venkataramani, A., 2006, ‘Technical efficiency in agricultural production and its determinants: An exploratory study at the district level’, *Indian Journal of Agricultural Economics* 61(2), 169–184.

[CIT0036] Swart, C., 2016, *Dialogue. Drought 2015 and 2016. Challenges facing commercial producers*, AGRI Western Cape, viewed 12 October 2019, from https://www.greenagri.org.za/assets/documents-/Drought-dialogue-2016-/Cornie-Swart-Agri-Western-Cape-Presentation.pdf.

[CIT0037] UNDP, 2007, *Population Division of the Department of Economic and Social Affairs of the United Nations Secretariat, world population prospects: The 2006 revision and world urbanization prospects, 2005 revision*, viewed 24 June 2018, from http://esa.un./un./unpp.

[CIT0038] Vermeulen, S.J., Aggarwal, P.K., Ainslie, A., Angelone, C., Campbell, B.M., Challinor, A.J. et al., 2010, *Agriculture, food security and climate change: Outlook for knowledge, tools and action*, CCAFS Report 3, CGIAR Research Program on Climate Change, Agriculture and Food Security, Copenhagen.

[CIT0039] Zwane E.M., 2019, ‘Impact of climate change on primary agriculture, water sources and food security in Western Cape, South Africa’, *Jàmbá: Journal of Disaster Risk Studies* 11(1), a562. 10.4102/jamba.v11i1.562 PMC6489149 31049161

